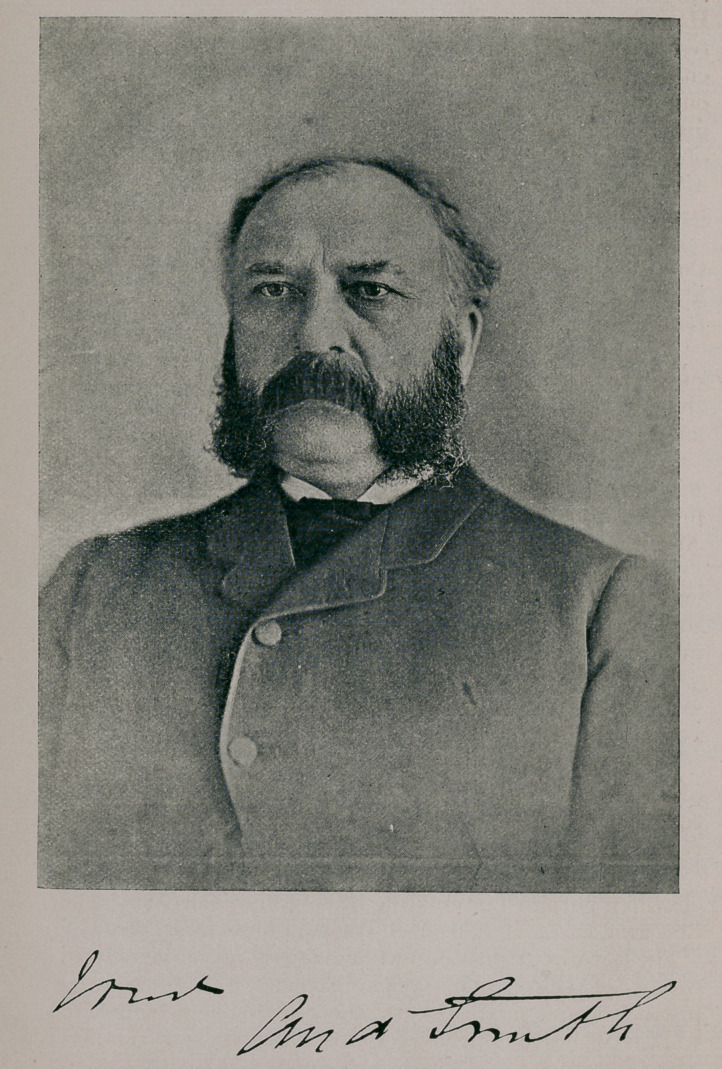# Ontario Veterinary College, Toronto, Canada

**Published:** 1888-10

**Authors:** 


### ARTICLE XXIV—ONTARIO VETERINARY COLLEGE, TORONTO, CANADA.

About the year 1859 members of the Board of Agriculture for Upper Canada, became convinced that some steps must be taken to provide veterinary instruction for young men. The rapid progress of the country, the great development of its farming and stock interests, with the absence of educated veterinarians, rendered the necessity for education in veterinary matters urgent.

Among the members of the Board who were more particularly impressed with the importance of the above-named

Yours
Wm A Smith subject, were the late Hon. Adam Fergusson, the late Hon. David Christie and the late Professor Buckland. Professor Buckland was in charge of the Department of Agriculture at this time, and his idea was to obtain a thoroughly competent graduate of a British college, who might give lectures and instruction to the classes on the diseases of farm animals, with the ultimate prospect of establishing a Veterinary College. In the pursuit of this design, Professor Buckland visited England in 1860.

Professor Dick was then at the head of the Edinburgh College, and application was made to him to recommend a graduate who might be able to fulfill the onerous duty of founding a veterinary college in America.

After giving the matter the consideration which its importance demanded, Professor Dick recommended a gentleman who had recently graduated, and who, he thought, gave promise of a brilliant career—Mr. Andrew Smith.

Professor Buckland at once opened up a correspondence with Mr. Smith, and fortunately for the veterinary profession in the New World, succeeded in inducing him to cross the Atlantic, and settle in Toronto. This was in the year 1861.

In the winter of 1862 the first course of lectures to Agricultural students was given, and those gentlemen who had so earnestly labored for this, had the greatest satisfaction in this consummation of their plans. In the two following years lectures were given as in 1862, but it then became evident that there was a demand for the establishment of a veterinary school or college.

In 1865, the plans of Professor Smith having been approved by the Board of Agriculture, regular courses of lectures on veterinary subjects were given, and the foundation of the Ontario Veterinary College thus laid.

The first session was attended by four or five students, all of whom purposed graduating in veterinary surgery. The first graduating class from the school numbered three.

The lectures at this time were given in Agricultural Hall, corner of Queen and Yonge Streets, while for clinical instruction and practical work, the students were obliged to walk a considerable distance to the Infirmary, on Temperance Street. In spite of these inconveniences, however, the students enthusiastically pursued their studies.

The members of the staff at this time were not numerous, but valuable assistance was given by Dr. Bovell, who lectured on physiology, and by J. J. Meyrick, V.S , to the Artillery Corps of the British Army, then stationed in Toronto. W. McEachran, V.S. of Woodstock, a fellow graduate and friend of Mr. Smith, also gave some lectures on Materia Medica.

The demand for professional men in the veterinary world of America, and the success of the graduates such, that there was a steady increase in the number of students attending. To accommodate them a new building was erected on Temperance Street, in connection with the Infirmary already there. This was in 1869, and were then about forty students in the classes. In 1876 the building was enlarged, a large room for a museum, as well as other rooms, being added. In 1881 the classes had outgrown the old lecture room, and the museum was utilized as a lecture room. After using this for four years, it, in its turn, was found too small, and a large hall was taken, which would easily seat three hundred and fifty persons. But last year, this again was found insufficient, and a hall which will seat four hundred and twenty persons was taken. In view of possible overcrowding even there, however, the early erection of large new buildings is contemplated. These new buildings would have been probably in use this session, but that labor troubles interfered with their commencement.

Of the members of the teaching staff, Professor Croft, Professor Buckland, Dr. Bovell and Dr. Barrett are no longer living. Mr. Meyrick is in London, England, where he holds an important position in the Veterinary Department of the British Army. He has recently been created a companion of the Bath. J. Thorburn, M D., is the oldest of the Professors now in charge. He was appointed to the chair of Materia Medica in 1869.

Dr. Barrett, took the chair of Physiology in 1872, and lectured up till his decease, which took place some two years ago.

Professor Buckland had the Department of the Breeding and Management of Farm Stock from the inception of the school till his death.

J. T. Duncan M.D., V.S., took the professorship of Anatomy in 1877, and is still in charge of that department; also that of Entozoa.

Gordon Richardson is Professor of Chemistry, and was appointed in 1885.

J. Cavan, M.D., takes Pathological Anatomy and Histology, having been appointed in 1887.

G. Peters, M.D., follows the lamented Dr. Barrett in the chair of Physiology, taking it first in 1887.

Able instructors assist in these various departments when necessary.

The course of instruction consists of two sessions of two terms each, although many students voluntarily take a third session. Students are also required to spend most of the summer months between the sessions in practising the profession, under the instruction of a qualified veterinary surgeon. This is a regulation that has been found of the greatest benefit; so much so, that some of the British colleges have adopted this regulation.

The whole course of instruction is made as practical as possible. All the lectures are prepared with a special view to the needs of the veterinary student, to assist him in his life work, viz., the practice of his profession. But while the teaching is thoroughly practical, the study of the theory of veterinary medicine and allied subjects is by no means neglected, but receives full attention. The thorough adaptation of the course of instruction to the needs of the public is attested by the success in practice of the graduates of the college.

It is attested in another way, viz., by the steady increase in the number of students attending, its graduating class numbering, in 1866, three; in 1888, one hundred and twenty-five. And this success has been accomplished practically without State aid or any other extraneous influence in its favor.

Professor Smith, the founder and Principal of the college, is a native of Dalrymple, Ayrshire, Scotland, and his father, the late Mr. James Smith, was long well known as an enterprising and highly respected farmer. Mr. Andrew Smith studied his profession under the late Prof. Dick, of Edinburgh, and graduated with highest honors, having during his course of study gained four medals, beside other prizes from the Highland and Agricultural Society of Scotland, embracing anatomy, chemistry, and the best general examination on all subjects. He was also awarded the silver medal for highest honors in Dr. Stevenson Macadam's class at the Royal College of Surgeons. He was Secretary to the Dalrymple Farmers' Society for several years previous to beginning studies, and Secretary to the Edinburgh Veterinary Medical Society for the session of 1860–61, elected by an unanimous vote.

Mr. Smith, as well as being an accomplished veterinarian, is also considered one of the best horsemen in Canada, and has shown his judgment in the selection of stock, by the importation of several of our finest thoroughbreds.

Prof. Smith may justly be termed the pioneer of the veterinary profession in this country, as by his energy and perseverance as a teacher, and by his success and straightforwardness as a practitioner he has done much to elevate the profession in Canada, and to bring it to the position which it occupies to-day.

Professor Smith is honorary President of the Ontario Veterinary Medical Society, Past President of the Caledonian Society of Toronto, Past Master of St. Andrew's Lodge, No. 16, A. F. and A. M., of Canada; Director of the Ontario Industrial Exhibition Association; an honorary Foreign Associate of the Royal College of Veterinary Surgeons, and a Fellow by examination of the same body.

## Figures and Tables

**Figure f1:**